# ABOVE: cerclage after caesarean: protocol for a randomised controlled trial to assess the optimal preventative management for preterm birth secondary to caesarean section damage

**DOI:** 10.1186/s12884-026-08816-9

**Published:** 2026-02-20

**Authors:** Laura van der Krogt, Jenny Carter, Giorgia Dalla Valle, Natalie Suff, Lisa Story, Rachel M. Tribe, Andrew Shennan

**Affiliations:** Department of Women and Children’s Health, School of Life Course and Population Sciences, King’s College London, 10th Floor, North Wing, St Thomas’ Hospital, Westminster Bridge Road, London, SE1 7EH UK

**Keywords:** Preterm birth, Miscarriage, Mid-trimester loss, Caesarean section, Full dilatation, In-labour caesarean, Cerclage, Transabdominal cerclage, Transvaginal cerclage, Cervical caesarean damage

## Abstract

**Background:**

There is emerging evidence that caesarean section in labour is associated with an increased risk of recurrent mid-trimester loss (MTL) and spontaneous preterm birth (sPTB) in subsequent pregnancies. This is likely due to inadvertent damage to the cervical tissue at the time of caesarean section. Transvaginal cerclage (TVC) has been demonstrated to be less successful in this high-risk cohort of women. However, transabdominal cerclage (TAC) may be more effective as the suture is placed above the level of cervical caesarean damage. A TAC can be placed before or during early pregnancy, although placement during pregnancy may be less effective. To determine the optimal clinical management for women with this risk factor, the ABOVE trial will evaluate the effectiveness of TAC compared to TVC in women who have experienced a MTL or sPTB following an in-labour caesarean section.

**Methods:**

ABOVE is a multicentre randomised control trial comparing TAC or TVC as a preventative strategy for sPTB in women with history of an in-labour CS and subsequent MTL (14^+ 0^ -23^+ 6^ weeks) or sPTB (< 30 weeks). Participants will be allocated to one of two groups - Group A (currently pregnant < 14 weeks’ gestation) and Group B (planning a pregnancy) - and randomised 1:1 to TAC or TVC within each group.

**Discussion:**

The ABOVE trial will investigate the efficacy of known interventions in the prevention of preterm birth, TVC and TAC, in women who have had a previous in-labour CS and subsequent MTL or sPTB. The trial will inform the development of evidence-based practice guidelines to optimise the care offered to this cohort of high-risk women. With escalating rates of CS and the increased recognition of the relationship between caesarean cervical damage and sPTB and MTL, this work is key.

**Trial registration:**

ISCRTN 10977996 (17/05/2024).

**Supplementary Information:**

The online version contains supplementary material available at 10.1186/s12884-026-08816-9.

## Background

There is increasing evidence that prior pregnancies ending with delivery by in-labour caesarean (CS) are associated with both spontaneous preterm birth(sPTB), i.e. delivery < 37 weeks completed gestation, and mid-trimester loss (MTL), i.e. pregnancy loss between 14 and 24 weeks, in subsequent pregnancies. A retrospective analysis of US cohort data demonstrated that full dilatation CS (FDCS) is associated with a six-fold increased risk of sPTB compared to first stage CS [1]. Adding to this concern is the high risk of a recurrent sPTB in this group of women [2, 3]. A recent study reported that the relative risk (RR) of recurrent sPTB at < 37 weeks gestation is 2.7 for all emergency caesarean sections (EMCS) and 3.1 for a full dilatation CS (FDCS), when compared to women with a prior sPTB and without EMCS as a risk factor [2]. This risk further increases when including MTL as an outcome, with 25.4% delivering at < 24 weeks of gestation (RR 5.65) [2]. Another study demonstrated a 41% absolute risk of recurrence of sPTB at < 30 weeks of gestation [3].

The relationship between sPTB, MTL and in-labour caesarean may be related to inadvertent cervical damage at the time of CS (cervical caesarean damage, CCD). At the time of in-labour caesarean, the surgical incision itself, or surgical site extensions, may be near or within the cervical tissue. This could disrupt the integrity of cervical tissue, leading to a cervical scar defect and resulting in compromised cervical function in future pregnancies [4]. The extent of CCD may also be influenced by the suture material at the time of caesarean section, the healing process and incidence of post-operative infection.Treatment modalities to reduce sPTB are currently limited but include cervical cerclage insertion [5]. There are three options for prophylactic suture placement: low vaginal or high vaginal cervical sutures (known as transvaginal cerclages - TVCs) or transabdominal cerclage (TAC). TVCs are placed during pregnancy, while TACs, placed at the level of the internal os, require laparotomy or laparoscopy either prior to pregnancy or in pregnancy [6]. In the UK, TACs are usually reserved for women who have had a trachelectomy or a failed vaginally placed cerclage, where they are known to be highly efficacious [6].

Treatment modalities to reduce sPTB are currently limited but include cervical cerclage insertion [5]. There are three options for prophylactic suture placement: low vaginal or high vaginal cervical sutures (known as transvaginal cerclages - TVCs) or transabdominal cerclage (TAC). TVCs are placed during pregnancy, while TACs, placed at the level of the internal os, require laparotomy or laparoscopy either prior to pregnancy or in pregnancy [6]. In the UK, TACs are usually reserved for women who have had a trachelectomy or a failed vaginally placed cerclage, where they are known to be highly efficacious [6].

TVCs are widely used to prevent sPTB in high-risk women [6]. However, retrospective studies show that they are ten-fold less effective in women who have had a sPTB following an in-labour CS, compared to other high-risk groups, with failure rates of 46% [4]. Conversely, TAC has a success rate of > 90% in high-risk women [7]. Another retrospective cohort study demonstrated that TAC was associated with less sPTB at < 30 weeks compared to TVC [8]. Moreover, women with a history of preterm birth and mid-trimester pregnancy loss report having a TAC in place reassuring [9].

Efficacy of TAC is thought to be related to the position of the suture above the scar. In pregnancy this may not be possible due to the implantation site and the inherent dangers of inserting a suture near the embryonic sac. Furthermore, increased vascularity in the pregnant cervix and an enlarged gravid uterus can make suture placement difficult. To date, there has been no comparison of outcomes from preconception and in-pregnancy TAC procedures.

In response to the accumulating, but often retrospective, evidence associating in-labour CS with MTL and sPTB, we have designed the ABOVE randomised control trial (RCT) to address this by comparing the effectiveness of TAC versus TVC in women who have had a previous sPTB or MTL following in-labour caesarean, both before and during pregnancy.

### Aim

The aim of this RCT is to reduce mid-trimester pregnancy loss and preterm birth in women with experience of MTL/sPTB after in-labour CS. The objectives of this study are:

1. To evaluate the effectiveness of TACs in comparison with TVCs in women whohave experienced a MTL/sPTB following in-labour caesarean section
2. To compare pregnancy outcomes in women who had a TAC placed prior topregnancy with those where a TAC was placed in early pregnancy

### Design

ABOVE is a multicentre RCT comparing TAC and TVC as a preventative strategy for sPTB in women with history of an in-labour CS and subsequent MTL (between 14^+ 0^-23^+ 6^ weeks’ gestation) or sPTB (< 30 weeks’). Participants will be allocated to one of two groups - Group A (already pregnant) and Group B (planning a pregnancy) - and randomised 1:1 to TAC or TVC within each group. It is important to separately evaluate pre-conception and in-pregnancy TACs, as the suture can be placed higher pre-pregnancy and this may provide better support around cervical weakness caused by CCD. Therefore, the trial is separately powered for these two groups, and will require complete data from 160 participants (*n* = 80 in Group A, *n* = 80 in Group B).

### Participant recruitment

The target is to recruit over a period of 18 months. Potential participants will be identified from women referred to obstetricians or specialist preterm birth services who are based in one of the participating sites. These will be NHS hospitals in the UK (please refer to the ISRCTN record for participating sites). Eligible potential participants will be initially approached by clinicians when they attend for post-delivery debrief, pre-pregnancy counselling or specialist preterm service appointments. They will be given verbal information about the study. If willing, they will be contacted by the local research team, which may include attending clinicians and/or research midwives, who will provide the written information sheet and the opportunity to ask questions. Potential participants will be given sufficient time to consider taking part with the expectation that a decision will be made within a timeframe that will allow the arrangement of the allocated intervention to be carried out before they are 14^+ 0^ weeks pregnant. If a potential participant does not speak or understand English, a professional interpreter will be utilised to ensure all eligible women are given the option to participate and informed consent is obtained.

Group A: (in-pregnancy recruitment): eligible participants will be referred by clinical staff in the first trimester, e.g., first appointment with preterm specialist, and will be contacted soon afterwards by clinical research midwives.

Group B: (pre-conception recruitment): eligible participants will be identified by referral (with the woman’s permission) to the research team by clinical staff following a MTL or sPTB < 30 weeks’. These potential participants will then be approached by research midwives shortly after their NHS bereavement or post-partum debriefing consultant appointment, which is usually carried out six weeks after the event, or preconception counselling appointment which may happen at any time before a future pregnancy.

### Inclusion and exclusion criteria

Women will be eligible for the trial if they:

- are willing and able to give informed consent
- are aged 16 or over
- have had a previous in-labour caesarean section (between 4 cm and 10 cm (fully dilated) followed by a MTL (> 14 w) or preterm birth (< 30 weeks)
- are pregnant, but will be less than 14 + 0 weeks’ gestation at time of allocated intervention (Group A) OR
- are not yet pregnant but are considering a further pregnancy (Group B)

Potential participants will not be eligible for the trial if they:

- are more than 14^+ 0^ weeks pregnant at time of randomisation (as insertion of TAC is associated with higher risk beyond this gestation)
- already have a cerclage or (Arabin) pessary in situ
- are not planning another pregnancy
- have a history of preterm birth (spontaneous/iatrogenic) prior to the in-labour caesarean section
- are pregnant and expecting more than one baby (multiple pregnancy)

### Randomisation and minimisation

Following written informed consent, participants (in either Group) will be randomised (1:1) to either TAC or TVC using a computer-generated randomisation procedure incorporated within the ABOVE trial database.

Recruiters and trial co-ordinators will not have access to the randomisation sequence. Due to the nature of the interventions, the study is not blinded to the care providers or participants; they will be informed at time of recruitment to which arm they have been randomised.

To ensure a balance between groups, randomisation will take account of whether the participant’s previous in-labour CS was carried out at 4–9 cm or at full dilatation (minimisation). This is because current thinking is that a CS at full dilatation is likely to be more damaging than one carried out earlier in labour. If a potential participant is unsure of her cervical dilatation at previous in-labour CS, further details will be sought from the hospital where the operation took place and if uncertainty remains, a decision will be made within the study team as to whether to include her in the study and if so, in which category she should be placed for the purposes of minimisation.

### Schedule of assessments

Following randomisation, the allocated procedure will be arranged. Participants in Group A will have the cerclage (TAC or TVC) inserted prior to 14 + 0 weeks’ gestation. Group B participants allocated to TAC will have the procedure placed before their next pregnancy and those allocated to TVC will have it placed during their next pregnancy before 14 + 0 weeks’ gestation. TACs are performed as an open or laparoscopic procedure under either regional or general anaesthetic, and may require an inpatient stay of up to three days. TVCs will be performed at the participant’s local maternity unit with the insertion technique and anaesthesia according to clinician’s discretion and local practices. TACs will usually remain in situ (to support any future pregnancies) while TVCs are removed at around 37 weeks’ gestation. TACs are more specialised procedures and so they may be carried out in designated specialist units, as per local practice. The timeframe between randomisation and study procedure is flexible from site to site, depending on theatre list availability, although all procedures must be carried out before the participant is 14 + 0 weeks’ pregnant.

### Follow up procedures

There are no trial specific follow up procedures. All other pregnancy care and special preterm prevention surveillance will continue according to local clinical protocols.

### End of study definition

To allow time for collection of outcome data and analysis, end of study is defined as is defined as six months after the expected date of delivery of the last participant recruited.

### Trial flowchart

**Fig. 1:**
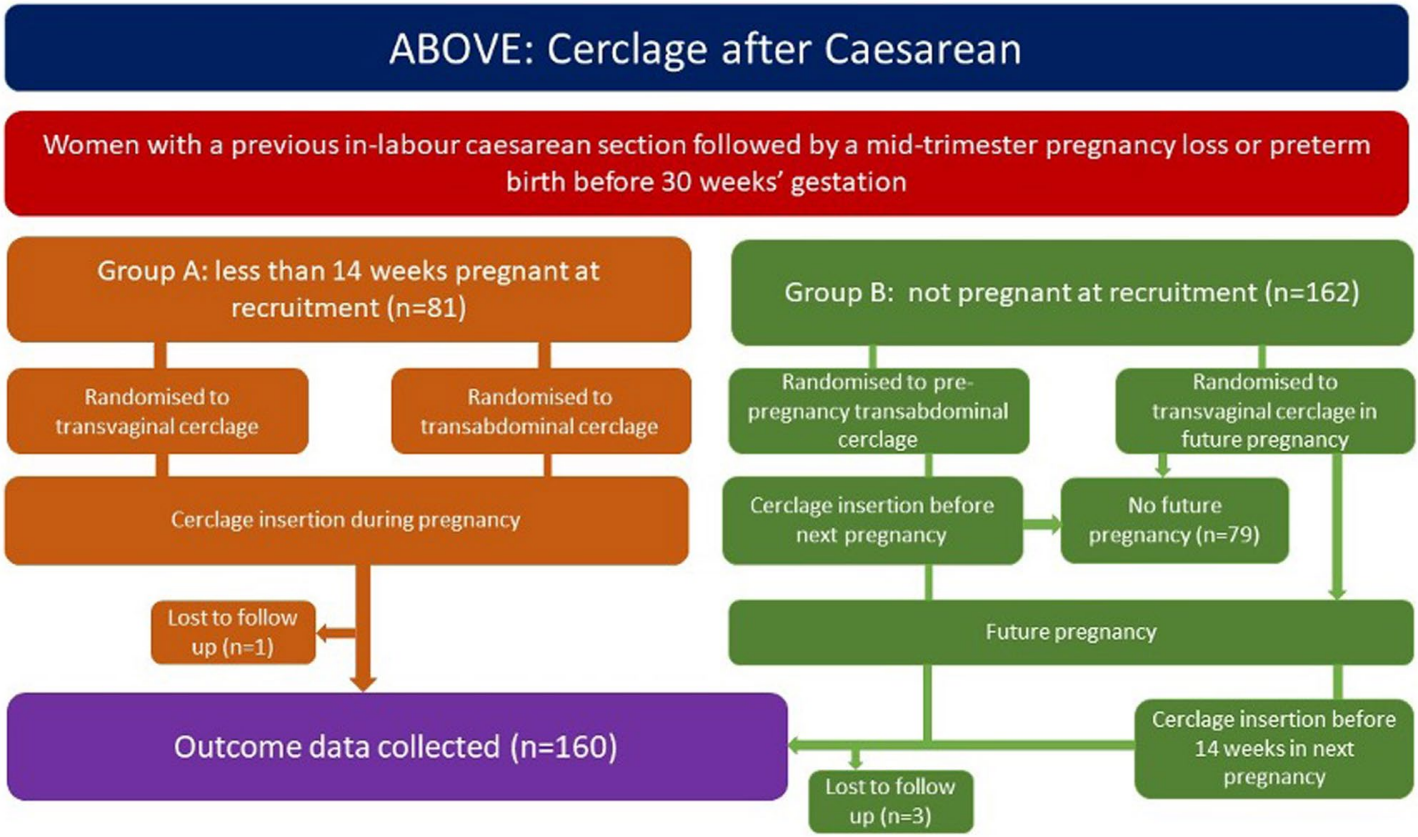
Trial flowchart (n=number)

See Fig. 1 for an illustration of participant flow through the study and anticipated number of participants.

### Outcome measures

#### Primary outcome

The occurrence of mid-trimester pregnancy loss or spontaneous preterm birth before 30 weeks of gestation.

#### Secondary outcomes

Maternal:

- Admission to hospital for symptoms of threatened preterm labour
- Administration of antenatal corticosteroids for fetal lung maturation
- Administration of magnesium sulphate for fetal cerebral protection
- Transfer to other hospitals for neonatal cot availability (*in utero* transfer)
- Time between intervention and delivery
- Requirement for additional emergency/rescue cerclage
- Serious complications occurring due to trial intervention: bladder injury, bowel injury, intraoperative rupture of membranes, cervical tear, hysterectomy
- Maternal sepsis
- Admission to ITU
- Maternal death

Neonatal:

- Gestation at birth
- Birthweight
- Apgar scores (if available)
- Days before discharge home (up to 28 days)
- Admission to neonatal unit
- Neonatal infection
- Any baby death

- ◦ *In utero* fetal death at or after 14 weeks
- ◦ Stillbirth
- ◦ Neonatal death

### Data collection

Data will be collected by attending clinician or research midwives and includes: at randomisation - participant background: demographic characteristics, risk factors, obstetric history; before pregnancy trial intervention procedure (Group B – TAC); during pregnancy - trial intervention procedures (Group A - TVC or TAC, Group B – TVC); clinical care: preterm surveillance methods and test results, e.g. infection screening, cervical length measurements, caesarean section scar characteristics, predictive biomarker tests, concomitant treatments; after pregnancy - outcomes: e.g. onset of labour, gestation at delivery, infection, admission to ITU (mother) and/or neonatal unit (baby).

Data will be stored on the ABOVE Trial Database, which is hosted within the already established PCN Database (www.medscinet.net/ukpcn). This is a secure web-based platform containing standardised clinical information regarding women at high risk of sPTB. The ABOVE database’s data monitoring facility will be used to ensure data quality [10]. This function allows efficient and remote raising and answering of queries by the central and site trial teams.

The data collected will allow us to evaluate the effectiveness of the interventions on preventing poor outcomes, whether there are differences between women from different groups (demographic characteristics and risk factors) and whether treatment effects are compounded by other variables, such as intensity of preterm surveillance and concomitant treatments.

### Data analysis

A data analysis plan has been prepared prior to commencement of study recruitment. Maternal, pregnancy, and surgical procedure characteristics will be presented with counts and percentages for categorical variables, mean and standard deviation for continuous Gaussian distributed variables, and median and interquartile range for continuous non-Gaussian variables.

All primary and secondary outcomes, and the components of the primary outcome will be summarised by treatment arm and Group (A or B) using frequencies and percentages.

In addition, relative risk will be calculated for the treatment effect (TAC vs. TVC) with 95% confidence intervals. An interaction test will be carried out to examine whether the treatment effect is significantly different between groups A or B. Where a difference is detected, separate relative risks will be presented for both groups. Where there is a significant treatment effect, Risk differences and Number Needed to Treat (NNT) will also be presented, with confidence intervals if significant. All these analyses will be corrected for the minimisation variable and Group (A or B).

The primary analyses will be by modified intention-to-treat for the pre-conception trial arms. Women who did not conceive, suffered an early miscarriage or are lost to follow-up will be excluded. A TVC will not be inserted in a non-pregnant patient (in line with standard clinical care) and therefore these participants will be removed from the trial, at the end of the 18-month recruitment period, so that the results relate to the number of stitches actually placed.

### Power calculation

A sample size estimation on data utilised data from a previous observational study performed by our group. This calculated that calculated that a TAC should provide a relative risk reduction of 67% for PTB < 30 weeks compared to TVC in this high-risk cohort of women (unpublished data). A total of 40 women in each intervention group (TAC/TVC) is required for 80% power, at 5% significance level, to show a significant difference between the two groups. This would equate to a total of 160 women in the study; 40 x group TVC vs. 40 x TAC in both groups: Group A (in pregnancy) and Group B (prior to pregnancy). The recruitment target accounts for a potential drop-out rate of 50% of Group B participants who may not have a subsequent pregnancy after randomisation; as informed by the MAVRIC trial [7].

### Trial steering committee

The role of the trial steering committee (TSC) is to provide the overall supervision of the study. The TSC will monitor the progress of the study and conduct and advise on its scientific credibility. The TSC will consider and act, as appropriate, upon the recommendations of the data monitoring committee (DMC) and ultimately carries the responsibility for deciding whether the trial needs to be stopped on the grounds of safety or efficacy. The TSC will consist of an independent chair and at least two other independent members (not involved in study recruitment and not employed by any organisation directly involved in study conduct). A representative from our dedicated preterm birth studies Patient Public Involvement group will be invited to participate. The first meeting will take place 6 months after trial start date; frequency will be decided at the first meeting (at least annually).

### Data monitoring committee

The DMC will be convened to ensure the wellbeing of study participants. The committee will periodically review study progress and outcomes as well as reports of serious adverse events (SAEs). The DMC will, if appropriate, make recommendations regarding the continuance of the study or modification of the study protocol. The DMC will meet 3 months following study commencement/recruitment of the first participant; frequency of meeting will be decided at the first meeting.

### Auditing and monitoring

The Chief Investigator will be responsible for the ongoing management of the study. The Sponsor will monitor and conduct audits on a selection of studies in its clinical research portfolio. Monitoring and auditing will be conducted in accordance with the UK Policy Framework for Health and Social Care and in accordance with the Sponsor’s monitoring and audit procedures. Protocol modifications will be communicated to relevant parties in line with UK research governance requirements. Adverse events will be reported as per regulations.

### Trial stopping rules

The ABOVE trial may be prematurely discontinued by the Sponsor, Chief Investigator or Regulatory Authority based on new safety information or for other reasons given by the DMC/TSC, regulatory authority or ethics committee concerned. If the trial is prematurely discontinued, active participants will be informed, and no further participant data will be collected. The Competent Authority and Research Ethics Committee will be informed within 15 days of the early termination of the trial.

### Insurance and indemnity

The study is co-sponsored by King’s College London (KCL) and Guys and St Thomas’ NHS Foundation Trust (GSTT). The sponsors will, at all times, maintain adequate insurance in relation to the study. KCL through its own professional indemnity (Clinical Trials) and no-fault compensation and GSTT having a duty of care to patients via NHS indemnity cover, in respect of any claims arising as a result of negligence by its employees, brought by or on behalf of a study participant.

### Dissemination policy

Results of the study will be presented at national and international conferences and reported in peer reviewed journals. No patient identifiable information will be published. A lay summary of the results will be presented back to the King’s College London/ Guy’s and St Thomas’ Preterm Birth Patient and Public Involvement Group, alongside being available for participants via the study website (https://www.medscinet.net/above).

Data will not be retained for use by other researchers (in a repository). This is because it will be impossible to adequately anonymise it due to the rarity of the condition and the small number of participants that will be recruited per site.

## Discussion

The emerging evidence demonstrating the association between recurrent sPTB and MTL with in-labour CS, compounded by escalating rates of CS globally, highlights the need to develop optimal management strategies for this high-risk cohort of women. This is even more important when considering that standard interventions for the prevention of sPTB, including TVC, may be less effective in this group. It is anticipated that the RCT design of the ABOVE study will provide useful evidence concerning the effectiveness of TACs in comparison with TVCs in this cohort and the impact of placing a TAC placed prior to pregnancy compared to in early pregnancy on pregnancy outcomes. This research will inform the development of evidence-based practice.

## Supplementary Information

Supplementary Material 1.

Supplementary Material 2.

Supplementary Material 3.

Supplementary Material 4.

Supplementary Material 5.

Supplementary Material 6.

## Data Availability

No datasets were generated or analysed during the current study.

## Abbreviations

CS: Caesarean Section
DMC: Data Monitoring Committee
EMCS: Emergency Caesarean Section
FDCS: Full Dilatation Caesarean Section
MTL: Mid Trimester Loss, pregnancy loss between 14 and 24 weeks’ gestation
PI: Principal investigator
RCT: Randomised Control Trial
REC: Research Ethics Committee
R&D: Research & Development
sPTB: Spontaneous Preterm Birth, delivery before 37 weeks completed gestation
TAC: Transabdominal Cerclage
TSC: Trial Steering Committee
TVC: Transvaginal Cerclage
