# Supplementary material for: ABOVE: cerclage after caesarean: protocol for a randomised controlled trial to assess the optimal preventative management for preterm birth secondary to caesarean section damage
Source: BMC Pregnancy Childbirth. 2026 Feb 20;26:336. doi: 10.1186/s12884-026-08816-9 (PMC13032483; doi:10.1186/s12884-026-08816-9)
Supplement: Supplementary file 1 — Supplementary Material 1. [file 12884_2026_8816_MOESM1_ESM.docx]

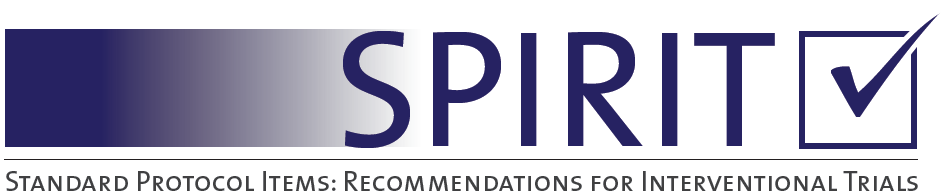


SPIRIT 2013 Checklist: Recommended items to address in a clinical trial protocol and related documents*

| Section/item | ItemNo | Description | Done? |
| --- | --- | --- | --- |
| **Administrative information** | | |  |
| Title | 1 | Descriptive title identifying the study design, population, interventions, and, if applicable, trial acronym | Yes included in manuscript – includes word ‘protocol’ and the study designs we are planning |
| Trial registration | 2a | Trial identifier and registry name. If not yet registered, name of intended registry | Yes included in manuscript (bottom of abstract) |
|  | 2b | All items from the World Health Organization Trial Registration Data Set | Yes – relevant ones answered and included in manuscript |
| Protocol version | 3 | Date and version identifier | Protocol version 1.0 dated 29^th^ February 2024 is in use currently  This is the first protocol version being published |
| Funding | 4 | Sources and types of financial, material, and other support | Yes included in manuscript (under declarations) |
| Roles and responsibilities | 5a | Names, affiliations, and roles of protocol contributors | Yes included in manuscript (under declarations) |
|  | 5b | Name and contact information for the trial sponsor | King’s College London and Guy’s and St Thomas’ are co-sponsors.  Kings College London  Professor Reza Razavi  Vice President & Vice Principal (Research)  King’s College London  Room 5.31, James Clerk Maxwell Building  57 Waterloo Road  London SE1 8WA  Telephone: +44(0) 207 8483224  Fax: 02071888330  Email: [reza.razavi@kcl.ac.uk](mailto:reza.razavi@kcl.ac.uk)  Guy’s and St Thomas’ NHS Foundation Trust  Elizabeth Bruna  Guy’s and St Thomas’ Foundation NHS Trust  R&D Department  16^th^ Floor Guy’s Hospital, Tower Wing,  Great Maze Pond  London SE1 9RT  Ext tel: 02071889811  Int [tel: 89811](tel:89811)  Fax: 02071881295  Email: [R&D@gstt.nhs.uk](mailto:R&D@gstt.nhs.uk) |
|  | 5c | Role of study sponsor and funders, if any, in study design; collection, management, analysis, and interpretation of data; writing of the report; and the decision to submit the report for publication, including whether they will have ultimate authority over any of these activities | Yes included in manuscript under declarations |
|  | 5d | Composition, roles, and responsibilities of the coordinating centre, steering committee, endpoint adjudication committee, data management team, and other individuals or groups overseeing the trial, if applicable (see Item 21a for data monitoring committee) | Yes included in manuscript under ‘Trial Steering Committee’ and ‘Data Monitoring Committee’ |
| Introduction |  |  |  |
| Background and rationale | 6a | Description of research question and justification for undertaking the trial, including summary of relevant studies (published and unpublished) examining benefits and harms for each intervention | Yes included in manuscript under ‘Background; |
|  | 6b | Explanation for choice of comparators | Yes included in manuscript under ‘Study Design’ |
| Objectives | 7 | Specific objectives or hypotheses | Yes included in manuscript under ‘Study Design’ |
| Trial design | 8 | Description of trial design including type of trial (eg, parallel group, crossover, factorial, single group), allocation ratio, and framework (eg, superiority, equivalence, noninferiority, exploratory) | Yes included in manuscript under ‘Study Design’ |
| Methods: Participants, interventions, and outcomes | | |  |
| Study setting | 9 | Description of study settings (eg, community clinic, academic hospital) and list of countries where data will be collected. Reference to where list of study sites can be obtained | Yes included in manuscript under ‘Study Design’ |
| Eligibility criteria | 10 | Inclusion and exclusion criteria for participants. If applicable, eligibility criteria for study centres and individuals who will perform the interventions (eg, surgeons, psychotherapists) | Yes included in manuscript under ‘Inclusion and Exclusion Criteria’ |
| Interventions | 11a | Interventions for each group with sufficient detail to allow replication, including how and when they will be administered | Yes included in manuscript under ‘Study Design’ |
|  | 11b | Criteria for discontinuing or modifying allocated interventions for a given trial participant (eg, drug dose change in response to harms, participant request, or improving/worsening disease) | Yes included in manuscript under ‘Randomisation and Minimisation’ |
|  | 11c | Strategies to improve adherence to intervention protocols, and any procedures for monitoring adherence (eg, drug tablet return, laboratory tests) | n/a |
|  | 11d | Relevant concomitant care and interventions that are permitted or prohibited during the trial | Yes, included in section “Follow up procedures”. Appropriate indications for withdrawal of treatment discussed in manuscript |
| Outcomes | 12 | Primary, secondary, and other outcomes, including the specific measurement variable (eg, systolic blood pressure), analysis metric (eg, change from baseline, final value, time to event), method of aggregation (eg, median, proportion), and time point for each outcome. Explanation of the clinical relevance of chosen efficacy and harm outcomes is strongly recommended | Yes in manuscript under ‘Outcome Measures’ |
| Participant timeline | 13 | Time schedule of enrolment, interventions (including any run-ins and washouts), assessments, and visits for participants. A schematic diagram is highly recommended (see Figure) | Yes in manuscript and in ‘Trial Flow Diagram’ |
| Sample size | 14 | Estimated number of participants needed to achieve study objectives and how it was determined, including clinical and statistical assumptions supporting any sample size calculations | Yes included in manuscript under ‘Power Calculation’ |
| Recruitment | 15 | Strategies for achieving adequate participant enrolment to reach target sample size | Yes discussed under sample size |
| **Methods: Assignment of interventions (for controlled trials)** | | |  |
| Allocation: |  |  |  |
| Sequence generation | 16a | Method of generating the allocation sequence (eg, computer-generated random numbers), and list of any factors for stratification. To reduce predictability of a random sequence, details of any planned restriction (eg, blocking) should be provided in a separate document that is unavailable to those who enrol participants or assign interventions | Yes included in manuscript under ‘Randomisation’  No planned restriction so this is not required to be included in a separate document |
| Allocation concealment mechanism | 16b | Mechanism of implementing the allocation sequence (eg, central telephone; sequentially numbered, opaque, sealed envelopes), describing any steps to conceal the sequence until interventions are assigned | Yes included in manuscript under ‘Randomisation and Minimisation’ |
| Implementation | 16c | Who will generate the allocation sequence, who will enrol participants, and who will assign participants to interventions | Yes included in manuscript under ‘Randomisation and Minimisation’ |
| Blinding (masking) | 17a | Who will be blinded after assignment to interventions (eg, trial participants, care providers, outcome assessors, data analysts), and how | Yes included in manuscript under ‘Randomisation and Minimisation’’. Due to the nature of the interventions, the study is not blinded to the care providers or patient. |
|  | 17b | If blinded, circumstances under which unblinding is permissible, and procedure for revealing a participant’s allocated intervention during the trial | n/a see above |
| **Methods: Data collection, management, and analysis** | | |  |
| Data collection methods | 18a | Plans for assessment and collection of outcome, baseline, and other trial data, including any related processes to promote data quality (eg, duplicate measurements, training of assessors) and a description of study instruments (eg, questionnaires, laboratory tests) along with their reliability and validity, if known. Reference to where data collection forms can be found, if not in the protocol | Yes included in manuscript under ‘Data Collection’ |
|  | 18b | Plans to promote participant retention and complete follow-up, including list of any outcome data to be collected for participants who discontinue or deviate from intervention protocols | Yes included in manuscript under ‘Data Collection’ |
| Data management | 19 | Plans for data entry, coding, security, and storage, including any related processes to promote data quality (eg, double data entry; range checks for data values). IReference to where details of data management procedures can be found, if not in the protocol | Yes included in manuscript under ‘Data Collection’ |
| Statistical methods | 20a | Statistical methods for analysing primary and secondary outcomes. Reference to where other details of the statistical analysis plan can be found, if not in the protocol | Yes included in manuscript under ‘Data Analysis’ |
|  | 20b | Methods for any additional analyses (eg, subgroup and adjusted analyses) | Yes included in manuscript under ‘Data Analysis’ |
|  | 20c | Definition of analysis population relating to protocol non-adherence (eg, as randomised analysis), and any statistical methods to handle missing data (eg, multiple imputation) | Yes included in manuscript under ‘Data Analysis’ |
| **Methods: Monitoring** | | |  |
| Data monitoring | 21a | Composition of data monitoring committee (DMC); summary of its role and reporting structure; statement of whether it is independent from the sponsor and competing interests; and reference to where further details about its charter can be found, if not in the protocol. Alternatively, an explanation of why a DMC is not needed | Yes in manuscript under ‘Data Monitoring Committee’ |
|  | 21b | Description of any interim analyses and stopping guidelines, including who will have access to these interim results and make the final decision to terminate the trial | Stopping guidelines - Yes in manuscript under ‘Trial Steering Committee’ and ‘Study Stopping Rules’. |
| Harms | 22 | Plans for collecting, assessing, reporting, and managing solicited and spontaneously reported adverse events and other unintended effects of trial interventions or trial conduct | Yes in manuscript under ~~safety~~ ‘Auditing and Monitoring’ |
| Auditing | 23 | Frequency and procedures for auditing trial conduct, if any, and whether the process will be independent from investigators and the sponsor | Yes in manuscript under ‘Auditing and Monitoring’ |
| Ethics and dissemination | | |  |
| Research ethics approval | 24 | Plans for seeking research ethics committee/institutional review board (REC/IRB) approval | Included in section ‘Ethics approval and consent to participate’. |
| Protocol amendments | 25 | Plans for communicating important protocol modifications (eg, changes to eligibility criteria, outcomes, analyses) to relevant parties (eg, investigators, REC/IRBs, trial participants, trial registries, journals, regulators) | Yes in section ‘Auditing and monitoring’. |
| Consent or assent | 26a | Who will obtain informed consent or assent from potential trial participants or authorised surrogates, and how (see Item 32) | Yes in manuscript under ‘Participant Recruitment’ and under declarations section ‘Ethics approval and consent to participate’ |
|  | 26b | Additional consent provisions for collection and use of participant data and biological specimens in ancillary studies, if applicable | n/a |
| Confidentiality | 27 | How personal information about potential and enrolled participants will be collected, shared, and maintained in order to protect confidentiality before, during, and after the trial | Yes in manuscript under ‘Data collection’ |
| Declaration of interests | 28 | Financial and other competing interests for principal investigators for the overall trial and each study site | Yes in manuscript under declarations |
| Access to data | 29 | Statement of who will have access to the final trial dataset, Iand disclosure of contractual agreements that limit such access for investigators | Yes in manuscript under ‘Dissemination Policy’ |
| Ancillary and post-trial care | 30 | Provisions, if any, for ancillary and post-trial care, and for compensation to those who suffer harm from trial participation | Yes in manuscript under ‘Insurance and Indemnity’ |
| Dissemination policy | 31a | Plans for investigators and sponsor to communicate trial results to participants, healthcare professionals, the public, and other relevant groups (eg, via publication, reporting in results databases, or other data sharing arrangements), including any publication restrictions | Yes in manuscript under ‘Dissemination Policy’ |
|  | 31b | Authorship eligibility guidelines and any intended use of professional writers | Yes and author contributions is in the manuscript under declarations |
|  | 31c | Plans, if any, for granting public access to the full protocol, participant-level dataset, and statistical code | Yes - the protocol has been made available on the study website – <https://www.medscinet.net/UKPCN/ABOVE/>  Yes - the details about the dataset access are in manuscript under ‘Dissemination Policy’ |
| Appendices |  |  |  |
| Informed consent materials | 32 | Model consent form and other related documentation given to participants and authorised surrogates | Yes Patient Information Leaflet and Consent form added as additional documentation in submission |
| Biological specimens | 33 | Plans for collection, laboratory evaluation, and storage of biological specimens for genetic or molecular analysis in the current trial and for future use in ancillary studies, if applicable | n/a |

*It is strongly recommended that this checklist be read in conjunction with the SPIRIT 2013 Explanation & Elaboration for important clarification on the items. Amendments to the protocol should be tracked and dated. The SPIRIT checklist is copyrighted by the SPIRIT Group under the Creative Commons “[Attribution-NonCommercial-NoDerivs 3.0 Unported](http://www.creativecommons.org/licenses/by-nc-nd/3.0/)” license.
