# Supplementary material for: ABOVE: cerclage after caesarean: protocol for a randomised controlled trial to assess the optimal preventative management for preterm birth secondary to caesarean section damage
Source: BMC Pregnancy Childbirth. 2026 Feb 20;26:336. doi: 10.1186/s12884-026-08816-9 (PMC13032483; doi:10.1186/s12884-026-08816-9)
Supplement: Supplementary file 2 — Supplementary Material 2. [file 12884_2026_8816_MOESM2_ESM.docx]

# **
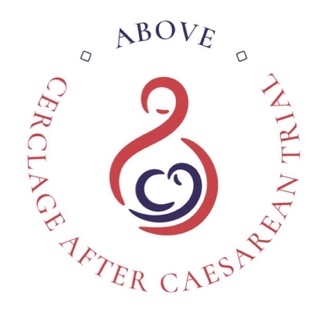
**Data Analysis Plan

## ABOVE: Cerclage After Caesarean

**General Considerations**

The statistical analyses and reporting will adhere to the Consolidated Standards of Reporting Trials (CONSORT)-guidelines (1,2). Stata (version 18 or later) will be used for data management and analyses.

Patient inclusion and exclusion will be illustrated in a modified CONSORT flow diagram for non-pharmacologic trials (3).

*CONSORT Flow Diagram of Inclusion, Treatment Allocation and Exclusions*


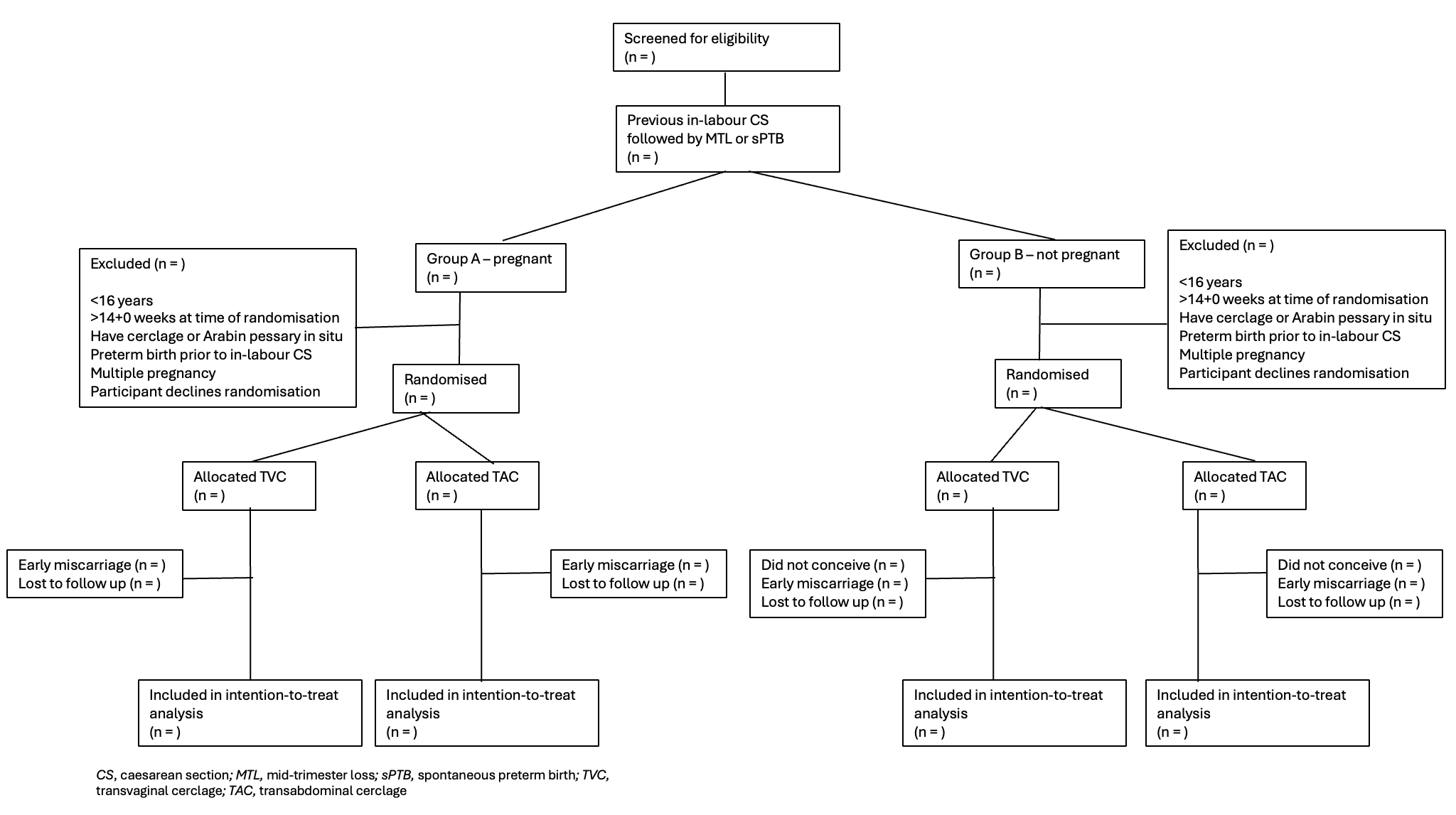


**Randomisation**

Following written informed consent, participants (in either Group) will be randomised

(1:1) to either TAC or TVC using a computer-generated randomisation procedure

incorporated within the ABOVE trial database. Recruiters and trial co-ordinators will not

have access to the randomisation sequence. Due to the nature of the interventions, the

study is not blinded to the care providers or participants; they will be informed at time of

recruitment to which arm they have been randomised.

**Sample Size**

We have based our sample size estimation on data from an observational study,

performed by our group, that showed TAC to have a relative risk reduction of 67% for PTB <30 weeks compared to TVC in this high-risk cohort of women (unpublished data). A total of 40 women in each intervention group (TAC/TVC) is required for 80% power, at 5% significance level, to show a significant difference between the two groups. This would equate to a total of 160 women in the study; 40 x group TVC vs. 40 x TAC in both groups: Group A (in pregnancy) and Group B (prior to pregnancy). The recruitment target takes into account a potential drop-out rate of 50% of Group B participants who may not have a subsequent pregnancy after randomisation; as informed by the MAVRIC trial (8). This power calculation has been calculated based on two arms of the trial (TVC vs TAC) and therefore, will allow sufficient power to detect a difference between TVC and TAC in each of the two groups.

**Baseline Characteristics**

Maternal, pregnancy, and surgical procedure characteristics will be presented with counts and percentages for categorical variables, mean and standard deviation for continuous Gaussian distributed variables, and median and interquartile range for continuous non-Gaussian variables.

**Table 1: Maternal baseline demographic characteristics**

|  | **Group A TVC**  **(n = )** | **Group A TAC**  **(n = )** | **Group B TVC**  **(n = )** | **Group B TAC**  **(n= )** | **All TVC**  **(n = )** | **All TAC (n = )** | **All**  **(n = )** | ***Definition*** |
| --- | --- | --- | --- | --- | --- | --- | --- | --- |
| Age at time of consent, years | Mean  (SD) | Mean  (SD) | Mean  (SD) | Mean  (SD) | Mean  (SD) | Mean  (SD) | Mean  (SD) |  |
| Body mass index, kg/m^2^ | Mean  (SD) | Mean  (SD) | Mean  (SD) | Mean  (SD) | Mean  (SD) | mean(  SD) | Mean  (SD) |  |
| Ethnicity   - Asian - Black - White - Other | n (%)  n (%)  n (%)  n (%) | n (%)  n (%)  n (%)  n (%) | n (%)  n (%)  n (%)  n (%) | n (%)  n (%)  n (%)  n (%) | n (%)  n (%)  n (%)  n (%) | n (%)  n (%)  n (%)  n (%) | n (%)  n (%)  n (%)  n (%) |  |

**Table 2: Cohort risk factors for spontaneous preterm birth by treatment allocation**

|  | **Group A TVC (n = )** | **Group A TAC (n = )** | **Group B TVC (n = )** | **Group B TAC (n = )** | **All TVC (n = )** | **All TAC (n = )** | **All**  **(n =)** | ***Definition*** |
| --- | --- | --- | --- | --- | --- | --- | --- | --- |
| Previous in-labour caesarean section   - 4-9cm - 10cm - Preceded by attempted instrumental delivery | n (%)  n (%)  n (%) | n (%)  n (%)  n (%) | n (%)  n (%)  n (% | n (%)  n (%)  n (% | n (%)  n (%)  n (% | n (%)  n (%)  n (% | n (%)  n (%)  n (%) |  |
| Previous cervical surgical procedures | n (%) | n (%) | n (%) | n (%) | n (%) | n (%) | n (%) | *Invasive surgical procedures on the cervix (LLETZ, LEEP, trachelectomy, knife cone)* |
| Congenital uterine malformation | n (%) | n (%) | n (%) | n (%) | n (%) | n (%) | n (%) | *Congenital uterine malformation (uterine didelphys, septate uterus, arcuate uterus)* |
| Smoking  (at time of recruitment) | n (%) | n (%) | n (%) | n (%) | n (%) | n (%) | n (%) | *Current smoker at time of recruitment* |
| Other medical and social risk factors   - Recurrent UTI (>3) in pregnancy - Group B streptococcus - Bacterial vaginosis - Recreational drug use - Domestic violence - Ehlers Danlos - Antiphospholipid syndrome - Late TOP - Index of multiple deprivation quintile | n (%)  n (%)  n (%)  n (%)  n (%)  n (%)  n (%)  n (%)  n (%) | n (%)  n (%)  n (%)  n (%)  n (%)  n (%)  n (%)  n (%)  n (%) | n (%)  n (%)  n (%)  n (%)  n (%)  n (%)  n (%)  n (%)  n (%) | n (%)  n (%)  n (%)  n (%)  n (%)  n (%)  n (%)  n (%)  n (%) | n (%)  n (%)  n (%)  n (%)  n (%)  n (%)  n (%)  n (%)  n (%) | n (%)  n (%)  n (%)  n (%)  n (%)  n (%)  n (%)  n (%)  n (%) | n (%)  n (%)  n (%)  n (%)  n (%)  n (%)  n (%)  n (%)  n (%) |  |

**Table 3: Characteristics of cerclage procedure**

|  | **Group A TVC (n = )** | **Group A TAC**  **(n = )** | **Group B TVC**  **(n = )** | **Group B TAC**  **(n = )** | **All TVC (n = )** | **All TAC (n = )** | ***Definition*** |
| --- | --- | --- | --- | --- | --- | --- | --- |
| Grade of surgeon   - Consultant - Senior registrar - Junior registrar - ST1-2 - Foundation doctor - Other - Unknown | n (%)  n (%)  n (%)  n (%)  n (%)  n (%)  n (%) | n (%)  n (%)  n (%)  n (%)  n (%)  n (%)  n (%) | n (%)  n (%)  n (%)  n (%)  n (%)  n (%)  n (% | n (%)  n (%)  n (%)  n (%)  n (%)  n (%)  n (% | n (%)  n (%)  n (%)  n (%)  n (%)  n (%)  n (% | n (%)  n (%)  n (%)  n (%)  n (%)  n (%)  n (% |  |
| Type of vaginal cerclage   - Shirodkar (with bladder mobilisation) - McDonald (without bladder mobilisation) | n (%)  n (%) | N/A | n (%)  n (%) | N/A | n (%)  n (%) | N/A |  |
| Timing of procedure   - Pre-pregnancy - During pregnancy | N/A  n (%) | n (%)  n (%) | N/A  n (%) | n (%)  n (%) | N/A  n (%) | n (%)  n (%) |  |
| Gestational age at placement, week and days | Mean (SD) | Mean (SD) | Mean (SD) | Mean (SD) | Mean (SD) | Mean (SD) |  |
| Anaesthesia   - General - Regional - Other | n (%)  n (%)  n (%) | n (%)  n (%)  n (%) | n (%)  n (%)  n (%) | n (%)  n (%)  n (%) | n (%)  n (%)  n (%) | n (%)  n (%)  n (%) |  |
| Cleaning   - Chlorhexidine based - Iodine based | n (%)  n (%) | n (%)  n (%) | n (%)  n (%) | n (%)  n (%) | n (%)  n (%) | n (%)  n (%) |  |
| Cervical characteristic   - Cervix normal - Cervix deficient anteriorly - Cervix deficient posteriorly - Other | n (%)  n (%)  n (%)  n (%) | n (%)  n (%)  n (%)  n (%) | n (%)  n (%)  n (%)  n (%) | n (%)  n (%)  n (%)  n (%) | n (%)  n (%)  n (%)  n (%) | n (%)  n (%)  n (%)  n (%) |  |
| Membranes visible | n (%) | n (%) | n (%) | n (%) | n (%) | n (%) | *Fetal membranes visualised at time of examination* |
| Catheterised pre-operatively | n (%) | n (%) | n (%) | n (%) | n (%) | n (%) | *Urinary catheter inserted prior to commencement of procedure* |
| Fluid for dissection   - No fluid dissection - Fluid dissection with vasoconstrictor - Fluid dissection with normal saline - Fluid dissection with water | n (%)  n (%)  n (%)  n (%) | n (%)  n (%)  n (%)  n (%) | n (%)  n (%)  n (%)  n (%) | n (%)  n (%)  n (%)  n (%) | n (%)  n (%)  n (%)  n (%) | n (%)  n (%)  n (%)  n (%) |  |
| Amount of fluid for dissection, mLs | Mean (SD) | Mean (SD) | Mean (SD) | Mean (SD) | Mean (SD) | Mean (SD) |  |
| Number of sutures   - 1 - 2 | n (%)  n (%) | n (%)  n (%) | n (%)  n (%) | n (%)  n (%) | n (%)  n (%) | n (%)  n (%) |  |
| Type of suture   - Monofilament 0 - Monofilament 1 - Other monofilament - Mersiline tape - Other tape | n (%)  n (%)  n (%)  n (%)  n (%) | n (%)  n (%)  n (%)  n (%)  n (%) | n (%)  n (%)  n (%)  n (%)  n (%) | n (%)  n (%)  n (%)  n (%)  n (%) | n (%)  n (%)  n (%)  n (%)  n (%) | n (%)  n (%)  n (%)  n (%)  n (%) |  |
| Suture tied   - Anteriorly - Posteriorly | n (%)  n (%) | n (%)  n (%) | n (%)  n (%) | n (%)  n (%) | n (%)  n (%) | n (%)  n (%) |  |
| Adjuvant Therapies Perioperatively   - Antibiotics - Thromboprophylaxis - NSAIDs - Progesterone - Other | n (%)  n (%)  n (%)  n (%)  n (%) | n (%)  n (%)  n (%)  n (%)  n (%) | n (%)  n (%)  n (%)  n (%)  n (%) | n (%)  n (%)  n (%)  n (%)  n (%) | n (%)  n (%)  n (%)  n (%)  n (%) | n (%)  n (%)  n (%)  n (%)  n (%) |  |
| Occlusion suture | n (%) | n (%) | n (%) | n (%) | n (%) | n (%) |  |
| Time under anaesthesia | Median (SD) | Median (SD) | Median (SD) | Median (SD) | Median (SD) | Median (SD) | *Time from start of anaesthetic to completion of procedure or patient awake* |
| Duration of surgery, minutes | Median (SD) | Median (SD) | Median (SD) | Median (SD) | Median (SD) | Median (SD) | *Time from start of operation to completion* |
| Blood loss, ml | Mean (SD) | Mean (SD) | Mean (SD) | Mean (SD) | Mean (SD) | Mean (SD) |  |
| No. of haemostatic sutures | Mean (SD) | Mean (SD) | Mean (SD) | Mean (SD) | Mean (SD) | Mean (SD) |  |
| Transfusion units, no. | Mean (SD) | Mean (SD) | Mean (SD) | Mean (SD) | Mean (SD) | Mean (SD) |  |
| Complications   - Bladder injury - Bowel injury - Intraoperative rupture of membranes - Hysterectomy - Unable to insert/remove suture - Cervical tear - ITU transfer - Maternal sepsis - Maternal death - Other | n (%)  n (%)  n (%)  n (%)  n (%)  n (%)  n (%)  n (%)  n (%)  n (%) | n (%)  n (%)  n (%)  n (%)  n (%)  n (%)  n (%)  n (%)  n (%)  n (%) | n (%)  n (%)  n (%)  n (%)  n (%)  n (%)  n (%)  n (%)  n (%)  n (%) | n (%)  n (%)  n (%)  n (%)  n (%)  n (%)  n (%)  n (%)  n (%)  n (%) | n (%)  n (%)  n (%)  n (%)  n (%)  n (%)  n (%)  n (%)  n (%)  n (%) | n (%)  n (%)  n (%)  n (%)  n (%)  n (%)  n (%)  n (%)  n (%)  n (%) |  |
| Catheterisation | Median (SD) | Median (SD) | Median (SD) | Median (SD) | Median (SD) | Median (SD) | *Number of days with urinary catheter in situ* |
| Length of inpatient stay, days | Median (SD) | Median (SD) | Median (SD) | Median (SD) | Median (SD) | Median (SD) |  |

**Statistical Analysis**

The primary outcome is a composite of any pregnancy loss between 14 and 30 weeks (mid-trimester pregnancy loss >14^+0^ weeks or spontaneous preterm birth <30^+0^ weeks). The predefined secondary outcomes are:

Maternal:

- Admission to hospital for symptoms of threatened preterm labour.
- Administration of antenatal corticosteroids for fetal lung maturation.
- Administration of magnesium sulphate for fetal celebral protection.
- Transfer to other hospitals for neonatal cot availability (*in -utero* transfer).
- Time between intervention and delivery.
- Requirement for additional emergency/rescue cerclage.
- Serious complications occurring as a result of trial intervention: bladder injury, bowel injury, intraoperative rupture of membranes, cervical tear, hysterectomy.
- Maternal sepsis.
- Admission to ITU.
- Maternal death.

Neonatal

- Gestation at birth.
- Birthweight.
- Apgar scores (if available).
- Days before discharge home (up to 28 days).
- Admission to neonatal unit.
- Neonatal infection.
- Any baby death
- *In utero* fetal death at or after 14 weeks.
- Stillbirth.
- Neonatal death.

All primary and secondary outcomes, and the components of the primary outcome will be summarised by treatment arm and Group (A or B) using frequencies and percentages.

In addition, relative risk will be calculated for the treatment effect (TAC vs TVC) with 95% confidence intervals. An interaction test will be carried out to examine whether the treatment effect is significantly different between groups A or B. Where a difference is detected, separate relative risks will be presented for both groups. Where there is a significant treatment effect, Risk differences and Number Needed to Treat (NNT) will also be presented, with confidence intervals if significant. All these analyses will be corrected for the minimisation variable (Table 4) and Group (A or B).

The primary analyses will be by modified intention-to-treat for the pre-conception trial arms. Women who did not conceive, suffered an early miscarriage or are lost to follow-up will be excluded. A TVC will not be inserted in a non-pregnant patient (in line with standard clinical care) and therefore these participants will be removed from the trial, at the end of the 18-month recruitment period, so that the results relate to the number of stitches actually placed.

**Table 4: Variables used for minimization by trial allocation after exclusions**

|  | **Group A TVC**  **(n = )** | **Group A TAC**  **(n = )** | **Group B TVC**  **(n = )** | **Group B TAC**  **(n = )** | **All TVC**  **(n = )** | **All TAC**  **(n = )** | ***Definition*** |
| --- | --- | --- | --- | --- | --- | --- | --- |
| Previous caesarean section at 10cm | n (%) | n (%) | n (%) | n (%) | n (%) | n (%) |  |

**Table 5: Pregnancy outcomes (ABOVE pregnancy)**

|  | **Group A TVC (n = )** | **Group A TAC**  **(n = )** | **Group B TVC**  **(n = )** | **Group B TAC**  **(n =)** | **All TVC (n = )** | **All TAC (n = )** | **All**  **(n = )** | ***Definition*** |
| --- | --- | --- | --- | --- | --- | --- | --- | --- |
| Mid-trimester loss or preterm delivery < 30 | n (%) | n (%) | n (%) | n (%) | n (%) | n (%) | n (%) | *Primary outcome* |
| Mid-trimester loss | n (%) | n (%) | n (%) | n (%) | n (%) | n (%) | n (%) | *Components of primary outcome* |
| Preterm delivery <30 weeks | n (%) | n (%) | n (%) | n (%) | n (%) | n (%) | n (%) | *Components of primary outcome* |
| Preterm delivery <34 weeks | n (%) | n (%) | n (%) | n (%) | n (%) | n (%) | n (%) |  |
| Preterm delivery <37 weeks | n (%) | n (%) | n (%) | n (%) | n (%) | n (%) | n (%) |  |
| Gestational age at pregnancy ending, weeks and days | Median (Quartiles) | Median (Quartiles) | Median (Quartiles) | Median (Quartiles) | Median (Quartiles) | Median (Quartiles) | Median (Quartiles) | *Descriptive only; not formal comparisons* |

**Table 6: comparisons between randomised treatments**

|  | **Risk Ratio with CI and P values**  **Primary comparison (groups A and B combined)** | **Risk difference**  **And NNT if RR significant** | **Interaction test between group A and group B** | **Risk Ratio with CI and P values**  **Primary comparison**  **(group A only, if needed)** | **Risk difference if RR significant** | **Risk Ratio with CI and P values**  **Primary comparison**  **(group B only, if needed)** | **Risk difference if RR significant** |
| --- | --- | --- | --- | --- | --- | --- | --- |
| Mid-trimester loss or preterm delivery < 30 | RR  (95% CI)  P= 0.XXX | RD  (95% CI)  P=0.XXX  NNT  (95% CI) | P=0.XXX | RR  (95% CI) | RD  (95% CI)  P=0.XXX  NNT  (95% CI) | RR  (95% CI) | RD  (95% CI)  P=0.XXX  NNT  (95% CI) |
| Mid-trimester loss | RR  (95% CI)  P= 0.XXX | RD  (95% CI)  P=0.XXX  NNT  (95% CI) | P=0.XXX | RR  (95% CI) | RD  (95% CI)  P=0.XXX  NNT  (95% CI) | RR  (95% CI) | RD  (95% CI)  P=0.XXX  NNT  (95% CI) |
| Preterm delivery <30 weeks | RR  (95% CI)  P= 0.XXX | RD  (95% CI)  P=0.XXX  NNT  (95% CI) | P=0.XXX | RR  (95% CI) | RD  (95% CI)  P=0.XXX  NNT  (95% CI) | RR  (95% CI) | RD  (95% CI)  P=0.XXX  NNT  (95% CI) |
| Preterm delivery <34 weeks | RR  (95% CI)  P= 0.XXX | RD  (95% CI)  P=0.XXX  NNT  (95% CI) | P=0.XXX | RR  (95% CI) | RD  (95% CI)  P=0.XXX  NNT  (95% CI) | RR  (95% CI) | RD  (95% CI)  P=0.XXX  NNT  (95% CI) |
| Preterm delivery <37 weeks | RR  (95% CI)  P= 0.XXX | RD  (95% CI)  P=0.XXX  NNT  (95% CI) | P=0.XXX | RR  (95% CI) | RD  (95% CI)  P=0.XXX  NNT  (95% CI) | RR  (95% CI) | RD  (95% CI)  P=0.XXX  NNT  (95% CI) |

**Table 7: Antenatal and delivery details (ABOVE trial pregnancy)**

|  | **Group A TVC**  **(n = )** | **Group A TAC**  **(n = )** | **Group B TVC**  **(n = )** | **Group B TAC**  **(n =)** | **All TVC (n= )** | **All TAC (n= )** | **All**  **(n = )** | ***Definition*** |
| --- | --- | --- | --- | --- | --- | --- | --- | --- |
| Concomitant treatment with vaginal progesterone | n (%) | n (%) | n (%) | n (%) | n (%) | n (%) | n (%) |  |
| Additional emergency/rescue cerclage | n (%) | n (%) | n (%) | n (%) | n (%) | n (%) | n (%) |  |
| Threatened mid-trimester loss or preterm labour | n (%) | n (%) | n (%) | n (%) | n (%) | n (%) | n (%) | *Admission to hospital for symptoms of threatened mid-trimester loss or preterm labour* |
| *In-utero* transfer | n (%) | n (%) | n (%) | n (%) | n (%) | n (%) | n (%) | *Transfer to other hospitals for neonatal cot availability* |
| Administration of tocolytics | n (%) | n (%) | n (%) | n (%) | n (%) | n (%) | n (%) |  |
| Administration of steroids | n (%) | n (%) | n (%) | n (%) | n (%) | n (%) | n (%) | *For fetal lung maturity* |
| Administration of magnesium sulphate | n (%) | n (%) | n (%) | n (%) | n (%) | n (%) | n (%) | *For fetal neuroprotection* |
| PPROM | n (%) | n (%) | n (%) | n (%) | n (%) | n (%) | n (%) | *Preterm pre-labour rupture of membranes* |
| Onset of labour   - Spontaneous onset of labour - Induction of labour - Caesarean section | n (%)  n (%)  n (%) | n (%)  n (%)  n (%) | n (%)  n (%)  n (%) | n (%)  n (%)  n (%) | n (%)  n (%)  n (%) | n (%)  n (%)  n (%) | n (%)  n (%)  n (%) |  |
| Mode of delivery   - Spontaneous vaginal - Assisted vaginal (forceps or ventouse) - Elective caesarean section - Emergency caesarean section prior to the onset of labour - Emergency caesarean section in labour | n (%)  n (%)  n (%)  n (%)  n (%)  n (%) | n (%)  n (%)  n (%)  n (%)  n (%)  n (%) | n (%)  n (%)  n (%)  n (%)  n (%)  n (%) | n (%)  n (%)  n (%)  n (%)  n (%)  n (%) | n (%)  n (%)  n (%)  n (%)  n (%)  n (%) | n (%)  n (%)  n (%)  n (%)  n (%)  n (%) | n (%)  n (%)  n (%)  n (%)  n (%)  n (%) |  |
| Interval between cerclage and delivery (weeks) | Mean  (SD) | Mean (SD) | Mean (SD) | Mean (SD) | Mean (SD) | Mean (SD) | Mean (SD) |  |
| Interval between cerclage and conception (weeks) | N/A | N/A | Mean (SD) | Mean (SD) | N/A | N/A | N/A |  |

**Table 8a : Neonatal outcomes (ABOVE trial pregnancy, singletons only)**

|  | **Group A TVC**  **(n = )** | **Group A TAC**  **(n = )** | **Group B TVC**  **(n=)** | **Group B TAC**  **(n=)** | **All TVC (n=)** | **All TAC (n=)** | **All**  **(n = )** | ***Definition*** |
| --- | --- | --- | --- | --- | --- | --- | --- | --- |
| Gestation at birth | Median (Quartiles) | Median (Quartiles) | Median (Quartiles) | Median (Quartiles) | Median (Quartiles) | Median (Quartiles) | Median (Quartiles) |  |
| Birthweight | Mean (SD) | Mean (SD) | Mean (SD) | Mean (SD) | Mean (SD) | Mean (SD) | Mean (SD) |  |
| APGAR scores  < 7   - 1 minute - 5 minutes - 9 minutes | n (%)  n (%)  n (%) | n (%)  n (%)  n (%) | n (%)  n (%)  n (%) | n (%)  n (%)  n (%) | n (%)  n (%)  n (%) | n (%)  n (%)  n (%) | n (%)  n (%)  n (%) |  |
| Days before discharge home   - 0-2 - 3-7 - 8-14 - 15-28 - >28 | n (%)  n (%)  n (%)  n (%)  n (%) | n (%)  n (%)  n (%)  n (%)  n (%) | n (%)  n (%)  n (%)  n (%)  n (%) | n (%)  n (%)  n (%)  n (%)  n (%) | n (%)  n (%)  n (%)  n (%)  n (%) | n (%)  n (%)  n (%)  n (%)  n (%) | n (%)  n (%)  n (%)  n (%)  n (%) | *Up to 28 days* |
| Admission to neonatal unit | n (%) | n (%) | n (%) | n (%) | n (%) | n (%) | n (%) |  |
| Neonatal infection | n (%) | n (%) | n (%) | n (%) | n (%) | n (%) | n (%) | *Positive culture for infection within 48 hours* |
| *In-utero* fetal death | n (%) | n (%) | n (%) | n (%) | n (%) | n (%) | n (%) | *>14^+0^ weeks gestation* |
| Stillbirth | n (%) | n (%) | n (%) | n (%) | n (%) | n (%) | n (%) | *>24^+0^ weeks where baby dies before or during delivery* |
| Neonatal death | n (%) | n (%) | n (%) | n (%) | n (%) | n (%) | n (%) | *Death in the first 28 days of life > 24^+0^ weeks of gestation* |

**Loss to follow up**

Loss to follow up is defined as no information on date of delivery or gestational age at birth.

**References**

1. Schulz KF, Altman DG, Moher D. CONSORT 2010 Statement: Updated guidelines for reporting parallel group randomised trials. BMC Med [Internet]. 2010 Mar 24 [cited 2024 Aug 30];8(1):1–9. Available from: https://bmcmedicine.biomedcentral.com/articles/10.1186/1741-7015-8-18

2. Moher D, Hopewell S, Schulz KF, Montori V, Gøtzsche PC, Devereaux PJ, et al. CONSORT 2010 explanation and elaboration: Updated guidelines for reporting parallel group randomised trials. International Journal of Surgery. 2012 Jan 1;10(1):28–55.

3. Barbour V, Bhui K, Chescheir N, Clavien PA, Diener MK, Glasziou P, et al. CONSORT Statement for Randomized Trials of Nonpharmacologic Treatments: A 2017 Update and a CONSORT Extension for Nonpharmacologic Trial Abstracts. Ann Intern Med [Internet]. 2017 Jul 4 [cited 2024 Aug 30];167(1):40–7. Available from: https://pubmed.ncbi.nlm.nih.gov/28630973/
