# Supplementary material for: ABOVE: cerclage after caesarean: protocol for a randomised controlled trial to assess the optimal preventative management for preterm birth secondary to caesarean section damage
Source: BMC Pregnancy Childbirth. 2026 Feb 20;26:336. doi: 10.1186/s12884-026-08816-9 (PMC13032483; doi:10.1186/s12884-026-08816-9)
Supplement: Supplementary file 4 — Supplementary Material 4. [file 12884_2026_8816_MOESM4_ESM.docx]

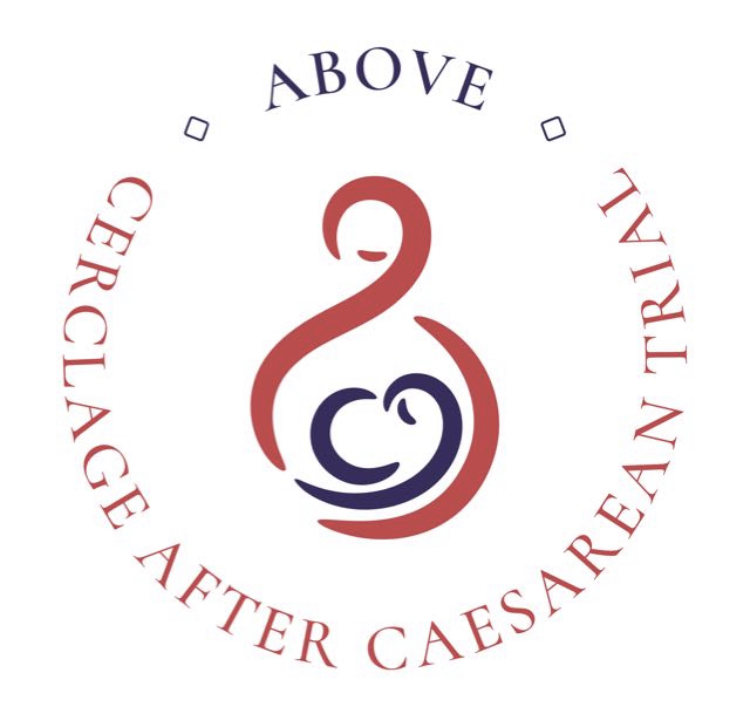


ABOVE ID:

B-####

**Cerclage after Caesarean: a randomised controlled trial to assess the optimal preventative management for preterm birth secondary to caesarean section damage (ABOVE)**

**Consent Form**

**Project Lead: Professor Andrew Shennan**

**Local Principal Investigator: ………….[***insert name***]……………….**

***Please initial in boxes***

1. I confirm that I have read and understand the information sheet (Group B - version # - ##/##/####) for the above project, have had the opportunity to ask questions and received satisfactory answers.
2. I understand that my participation is voluntary and that I am free to withdraw at any time, without giving a reason, without my medical care or legal rights being affected.
3. I give permission for the research team to access my medical records for the purposes of this research study.
4. I give permission for my personal information (including name, address, date of birth, telephone number and consent form) to be passed to King’s College London for administration of the study.

1. I understand that data collected will be stored on the study database and may be looked at by individuals from the Sponsor (KCL/GSTT), from the NHS organisation, affiliated collaborators, or authorities where it is relevant to my taking part in this project. I give permission for these individuals to have access to my records.
2. If I have my baby elsewhere, I am happy for the information about the birth to be sought from my GP and/or other hospitals.
3. I understand that data generated during the study will be sent outside of the United Kingdom where laws protecting my personal information may be different to my own country.
4. I agree to take part in the above study.

**Signatures**

___________________________ ____________________________ _________________

Name of Participant Signature Date

___________________________ ____________________________ _________________

Name of Person taking consent Signature Date
