# Supplementary material for: ABOVE: cerclage after caesarean: protocol for a randomised controlled trial to assess the optimal preventative management for preterm birth secondary to caesarean section damage
Source: BMC Pregnancy Childbirth. 2026 Feb 20;26:336. doi: 10.1186/s12884-026-08816-9 (PMC13032483; doi:10.1186/s12884-026-08816-9)
Supplement: Supplementary file 6 — Supplementary Material 6. [file 12884_2026_8816_MOESM6_ESM.docx]

**
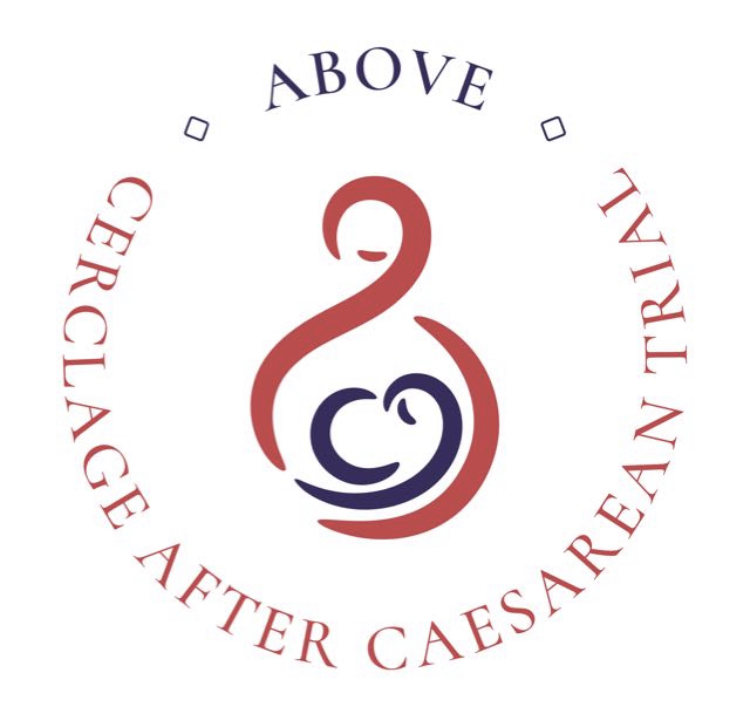
**

Trust logo

**Cerclage after Caesarean: a randomised controlled trial to assess the optimal preventative management for preterm birth secondary to caesarean section damage (ABOVE)**

**PARTICIPANT INFORMATION SHEET**

GROUP A (recruited in pregnancy)

**Invitation to take part**

This study is assessing whether a cerclage (a stitch placed around the cervix) is better if it is placed through the vagina, or abdominally (through a cut in the tummy) in women who have had a preterm birth or mid-trimester pregnancy loss (a miscarriage between 14 and 24 weeks of pregnancy) after a previous caesarean section in labour. Before you decide whether to participate, it is important for you to understand why the research is being done and what it will involve. Please take time to read the following information carefully and discuss it with friends, relatives and your hospital team if you wish. Ask us if there is anything that is not clear or if you would like more information. You will be given as long as you need to read the patient information sheet and consider participation.

**What is the purpose of this study?**

Recent studies have shown that if a woman has had a caesarean section in labour (when the cervix is opening) she is more likely to have a premature baby in a future pregnancy. We have found that in women who have had an in-labour caesarean section there is a 5-10% chance of a preterm birth in a subsequent pregnancy.


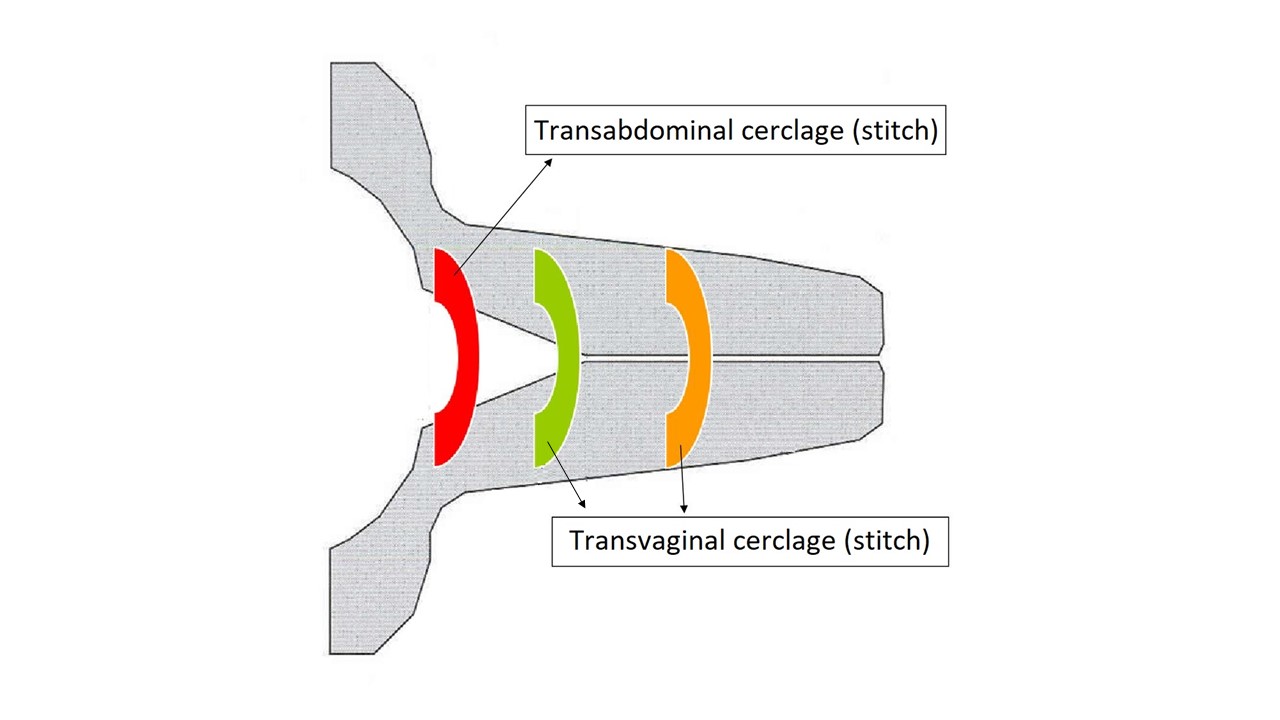
For women who have had an in-labour caesarean section, which was then followed by a preterm birth or mid-trimester loss, early birth is even more likely in subsequent pregnancies. Currently, we do not know which treatments are most effective to stop this happening. These women should be referred to specialist preterm clinics, who will offer them ultrasound monitoring of the length of their cervix, and they may or may not also be offered a cervical cerclage, although there are currently no national guidelines about this. This is a small surgical procedure where a stitch is placed around the cervix through the vagina (transvaginal cerclage). A cerclage can also be placed higher up, through an abdominal procedure involving a cut in the tummy (transabdominal cerclage). This procedure is a longer operation with more recovery time, and means that any future babies will need to be born by caesarean section. Both types of cerclage are offered as standard care to women at high risk of preterm birth. Although transvaginal cerclages are more straightforward, transabdominal cerclages might be more effective because they are above any damage that might have been caused during a previous caesarean section.

Figure: Placement positions of transabdominal and transvaginal cerclages.

**Why have I been asked to take part?**

You have been asked to take part because you are currently pregnant and have experienced a mid-trimester pregnancy loss or preterm birth after an earlier in-labour caesarean section.

**Do I have to take part?**

No, it is up to you to decide whether or not to take part. If you decide not to take part it will not affect the standard of medical care you receive.

**What will happen to me if I take part?**

If you do decide to take part you will be given this information sheet to keep and you will be asked to sign a consent form. You will be randomly allocated to one of two treatments: a transvaginal cerclage or a transabdominal cerclage. Whichever one you are allocated, it will performed before you are 14 weeks pregnant. You will have an equal chance of being allocated either of the two treatments, like the tossing of a coin. If you decide to take part you are still free to withdraw at any time and without having to give a reason.

Your experience will depend on which cerclage you have. The differences can be summarised as:

1. Transvaginal cerclage

If you are allocated the transvaginal cerclage it is usually inserted under regional anaesthetic (injection in the back) and you will therefore be awake. Women often go home the same day. The cerclage is removed when you are about 37 weeks pregnant, or earlier if you go into labour before then. You do not usually need an anaesthetic for the cerclage to be removed.

1. Transabdominal cerclage

If you are allocated the transabdominal cerclage it is usually inserted under a regional anaesthetic (injection in the back) and you will therefore be awake. If it is an “open” procedure, a small bikini-line cut will be made in your abdomen to place the cerclage. It may also be done by key-hole surgery. The procedure takes longer than a vaginal cerclage and you may have to stay in hospital for 2-3 days. The chance of damage to the bladder and bowel (uncommon risk occurring in 1 out of 1000) is very unlikely but is greater than with a vaginal cerclage. Your recovery time will also be slightly longer but most women fully recover within 2 weeks. You will need a caesarean section to deliver your baby and the cerclage will stay in place for future pregnancies, unless you want it removed at the time of caesarean (for example, if you know this will be your last pregnancy). Your fertility for future pregnancies is not affected.

With both stitches we would follow you up in the prematurity clinic with regular transvaginal scanning. This would be the same whether you were taking part in this study or not. If the cervix becomes short we may consider advising you to stay in hospital or offering you other treatments (such as a steroid injection to help your baby’s lungs mature, and/or putting in an additional stitch if the cervix opens). These may be offered if we think there is a high chance you will have your baby very early. Your care will be the same as it would if you do not take part in this study, apart from the addition of one of the two cerclages.

We will ask your permission to look at your medical notes and those of your baby after delivery so that we can find out what happened. If you have your baby in a different hospital we might need to contact your GP or the hospital where you have the baby. Although there are no study follow up visits, we will collect information about the care you receive during pregnancy, which may include scan images of your cervix taken in prematurity clinic.

**What are the disadvantages or side effects of taking part?**

Cervical cerclage is a relatively common procedure and is known to help some women at risk of preterm birth. The clinical teams carrying out the cerclages in this study are familiar and experienced with the procedures. The possible side effects and risks depend on which stitch you have.

Risks of both treatments include post-operative pain, infection and bleeding. The uncommon risks (occurring in 1 out of 100-1000) including tearing of the cervix or bladder,. Your obstetrician would generally be able to repair any tearing to the cervix immediately. A tear to the bladder would require another operation by a urologist (medical doctor with specialist training in problems of the urinary tract). Both would require a few extra days in hospital. If your doctor is worried about infection, a swab may be taken from your vagina and, if there is evidence of infection, you may be given a course of antibiotics.

While it is likely that either of the cerclages will reduce your chance of miscarriage or preterm birth, they will not entirely eliminate the possibility. The risk of miscarriage related to the procedure is low. If you have a transabdominal cerclage and you miscarry at less than 18 weeks, it should be possible to manage this without removing the cerclage. But if you miscarry after 18 weeks, you may need an operation to deliverthe baby. In addition to the above, an uncommon risk of inserting a transabdominal cerclage is damage to the bowel (occurring in 1 out of 1000). If this happens, a specialist surgeon will repair it. If you have a transabdominal cerclage, your baby will need to be born by caesarean section. A transabdominal cerclage does not appear to affect fertility.

More details can be found on the information sheets about the two types of cerclage provided by your local hospital.

**What are the possible benefits of taking part?**

Having either of the two cerclages will reduce your chance of having an early baby. However, taking part in the study may not have any direct benefit for your current pregnancy. What we learn from this study might help us to improve care for you in any future pregnancies as well as for other women, and reduce the number of babies being born too early.

**What if I change my mind after agreeing to take part?**

You are free to withdraw at any time without your medical care being affected. If you withdraw before you have had the cerclage placed, you should discuss your care options with the doctor (obstetrician) who is managing your pregnancy care. If you change your mind after the cerclage is placed we will not collect any further information but we will keep and use the information collected for the study so far.

**Further Supporting Information**

The information will be stored on the ABOVE trial database and ABOVE participant details database which are secure web-based platforms provided by MedSciNet, a Stockholm based company specialising in design and development of web applications and online database systems for clinical trials and studies. The data will be held on these databases, and downloaded to a KCL Sharepoint site for analysis. After completion of the study, research data it will be archived in a secure facility called Iron Mountain.

**Information on the use of data**

**How will we use information about you?**

We will need to use information from your hospital medical records for this research project. We will collect information on your previous medical history and pregnancies, your maternity care e.g. whether you had any other treatments relating to preterm birth, details about the birth e.g. onset of labour and how many weeks pregnant you were when the baby was born, and whether you and/or your baby had any problems or needed extra care e.g. admission to neonatal unit. We will collect this information during your pregnancy, e.g. whenever you attend specialist preterm clinic appointments, and after your baby has been born.

This information will include your initials, date of birth, hospital number and NHS number, but these identifiers will be kept on a separate but linked participant details database.  People will use this information to do the research or to check your records to make sure that the research is being done properly.

People who do not need to know who you are will not be able to see your name or contact details. Your data will have a code number instead.

We will keep all information about you safe and secure.

Some of your information will be sent to Sweden, where the servers for the main study and participant details databases are located. They must follow our rules about keeping your information safe. Appropriate safeguards will be in place, and the legal basis for storage of this data is that “it is a task in the public interest”.

Once we have finished the study, we will keep some of the data so we can check the results. We will write our reports in a way that no-one can work out that you took part in the study.

**What are your choices about how your information is used?**

- You can stop being part of the study at any time, without giving a reason, but we will keep information about you that we already have.
- We need to manage your records in specific ways for the research to be reliable. This means that we won’t be able to let you see or change the data we hold about you.

**Where can you find out more about how my information is used?**

You can find out more about how we use your information:

- at [www.hra.nhs.uk/information-about-patients/](https://www.hra.nhs.uk/information-about-patients/)
- our leaflet available from:
  [www.guysandstthomas.nhs.uk/research/patients/use-of-data.aspx](http://www.guysandstthomas.nhs.uk/research/patients/use-of-data.aspx) (For GSTT)
  and
  <https://www.kcl.ac.uk/research/research-environment/rgei/research-ethics/use-of-personal-data-in-research> (for KCL)
- by asking one of the research team (contact details included below)
- by contacting the Data Protection Officer: (For GSTT: Nick Murphy-O’Kane [DPO@gstt.nhs.uk](mailto:DPO@gstt.nhs.uk); For KCL: Olenka Cogias [info-compliance@kcl.ac.uk](mailto:info-compliance@kcl.ac.uk))

**What if there is a problem?**

If you have a concern about any aspect of this study, you should ask to speak to your local Principal Investigator who will do their best to answer your questions [*insert name and email address*]. If you remain unhappy and wish to complain formally, you can do this through your local hospital Patients Advice and Liaison Service (PALS) [*insert details*].

In the event that something goes wrong and you are harmed during the research you may have grounds for legal action for compensation against Guy’s and St Thomas’ NHS Foundation Trust and/or King’s College London but you may have to pay your legal costs. The normal National Health Service complaints mechanisms will still be available to you (if appropriate).

**What will happen to the results of the research study?**

The results will be published in the medical literature and presented at conferences. No participant names or identifiable information will be included. We will write a summary of the findings of the study and make it available on the study website (www.medscinet.net/ukpcn/above). Results will also be published through university, clinical and service user networks using email and social media.

**Who is organising and funding the research?**

Action Medical Research is funding the research, with sponsorship from King’s College London and Guy’s and St Thomas’ NHS Foundation Trust.

**Who has reviewed the study?**

This study has been reviewed and approved by this NHS Trust’s Research & Development Department. Health Research Authority and NHS Research Ethics Committee (REC Ref:#######; IRAS ID 327879).

**Contact for further information:**

*LOCAL Site information here*
